# First Detection of Bat White-Nose Syndrome in Western North America

**DOI:** 10.1128/mSphere.00148-16

**Published:** 2016-08-03

**Authors:** Jeffrey M. Lorch, Jonathan M. Palmer, Daniel L. Lindner, Anne E. Ballmann, Kyle G. George, Kathryn Griffin, Susan Knowles, John R. Huckabee, Katherine H. Haman, Christopher D. Anderson, Penny A. Becker, Joseph B. Buchanan, Jeffrey T. Foster, David S. Blehert

**Affiliations:** a U.S. Geological Survey, National Wildlife Health Center, Madison, Wisconsin, USA; b U.S. Forest Service, Northern Research Station, Center for Forest Mycology Research, Madison, Wisconsin, USA; c PAWS Wildlife Center, Lynnwood, Washington, USA; d Washington Department of Fish and Wildlife, Olympia, Washington, USA; e University of New Hampshire, Department of Molecular, Cellular and Biomedical Sciences, Durham, New Hampshire, USA; University of Wisconsin

**Keywords:** *Pseudogymnoascus destructans*, Washington, bat, white-nose syndrome

## Abstract

White-nose syndrome (WNS) is an emerging fungal disease of bats caused by *Pseudogymnoascus destructans*. Since it was first detected near Albany, NY, in 2006, the fungus has spread across eastern North America, killing unprecedented numbers of hibernating bats. The devastating impacts of WNS on Nearctic bat species are attributed to the likely introduction of *P. destructans* from Eurasia to naive host populations in eastern North America. Since 2006, the disease has spread in a gradual wavelike pattern consistent with introduction of the pathogen at a single location. Here, we describe the first detection of *P. destructans* in western North America in a little brown bat (*Myotis lucifugus*) from near Seattle, WA, far from the previously recognized geographic distribution of the fungus. Whole-genome sequencing and phylogenetic analyses indicated that the isolate of *P. destructans* from Washington grouped with other isolates of a presumed clonal lineage from the eastern United States. Thus, the occurrence of *P. destructans* in Washington does not likely represent a novel introduction of the fungus from Eurasia, and the lack of intensive surveillance in the western United States makes it difficult to interpret whether the occurrence of *P. destructans* in the Pacific Northwest is disjunct from that in eastern North America. Although there is uncertainty surrounding the impacts of WNS in the Pacific Northwest, the presence of the pathogen in western North America could have major consequences for bat conservation.

**IMPORTANCE** White-nose syndrome (WNS) represents one of the most consequential wildlife diseases of modern times. Since it was first documented in New York in 2006, the disease has killed millions of bats and threatens several formerly abundant species with extirpation or extinction. The spread of WNS in eastern North America has been relatively gradual, inducing optimism that disease mitigation strategies could be established in time to conserve bats susceptible to WNS in western North America. The recent detection of the fungus that causes WNS in the Pacific Northwest, far from its previous known distribution, increases the urgency for understanding the long-term impacts of this disease and for developing strategies to conserve imperiled bat species.

## Abstract (precis)

White-nose syndrome (WNS) represents one of the most consequential wildlife diseases of modern times. Since it was first documented in New York in 2006, the disease has killed millions of bats and threatens several formerly abundant species with extirpation or extinction. The spread of WNS in eastern North America has been relatively gradual, inducing optimism that disease mitigation strategies could be established in time to conserve bats susceptible to WNS in western North America. The recent detection of the fungus that causes WNS in the Pacific Northwest, far from its previous known distribution, increases the urgency for understanding the long-term impacts of this disease and for developing strategies to conserve imperiled bat species.

## Observation

**FIG 1:**
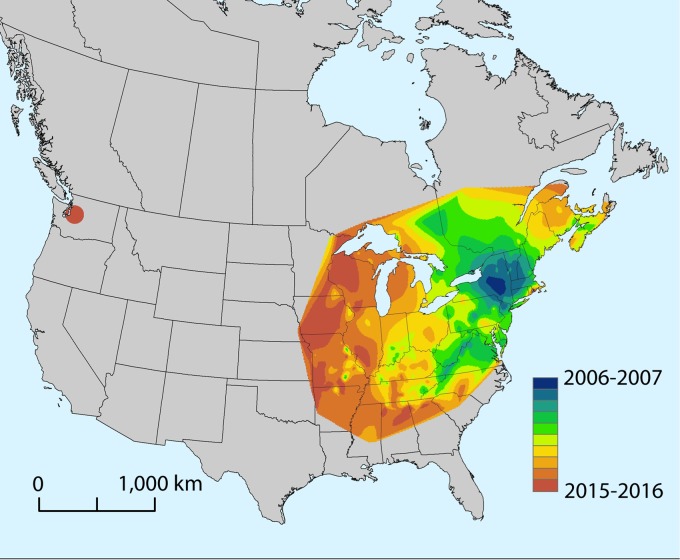
Generalized spatiotemporal spread of *Pseudogymnoascus destructans* across North America since the initial detection of white-nose syndrome (WNS) in New York in 2006. The map was generated using the natural-neighbor raster interpolation tool in ArcMap 10.2.1 (ESRI, Redlands, CA), based on first detection of *P. destructans* within a county or first classification of a county as suspect for WNS. The map was generated using data from the U.S. Geological Survey, National Wildlife Health Center, and https://www.whitenosesyndrome.org/resources/map. Each color represents the spread of the pathogen during a given winter (hibernation) season spanning from November to May.

White-nose syndrome (WNS) is a cutaneous infection of hibernating bats caused by the psychrophilic fungus *Pseudogymnoascus destructans* (1, 2). The disease, first documented in 2006, was subsequently associated with massive mortality of cave-hibernating bat species in the northeastern United States (3, 4). The emergence of WNS is likely due to the introduction of *P. destructans* into naive bat populations in North America. The fungus presumably originated in Eurasia, where it occurs on bats but is not known to cause severe infections or population declines (5–7). Consistent with a point source introduction of an exotic pathogen, *P. destructans* has spread outward from the WNS epicenter in New York by approximately 200 to 900 km per year (Fig. 1). As of spring 2016, the fungus had reached as far west as eastern Oklahoma, eastern Nebraska, and eastern Minnesota, approximately 1,900 km from the presumed site of introduction.

On 11 March 2016, a moribund little brown bat (*Myotis lucifugus*) was found in King County, WA (United States), and submitted to a local wildlife rehabilitation center. The animal presented with dried and contracted areas of crusted skin on the wings and died 2 days later. Swab samples of the wings were positive for *P. destructans* by real-time PCR (8), and the bat was confirmed to have WNS in accordance with defined histopathologic criteria (9). An isolate of *P. destructans* was obtained by culturing a portion of wing skin on Sabouraud dextrose agar containing chloramphenicol and gentamicin at 13°C.

In eastern North America, *P. destructans* appears to be spreading clonally, with all isolates exhibiting no genetic diversity at the markers examined (10). However, isolates of the fungus from Europe display significant genetic variation (11). To determine whether the isolate of *P. destructans* from Washington matched the clonal lineage from eastern North America, we conducted whole-genome sequencing using the Ion Torrent Personal Genome Machine (PGM) on the Washington isolate (NWHC#27099-001), as well as on three additional isolates of *P. destructans* from eastern North America. These isolates originated from *M. lucifugus* bats collected in Albany County, NY, in 2008 (NWHC#20631-008) and in Iowa County, WI, in 2016 (NWHC#26994-002) and a tri-colored bat (*Perimyotis subflavus*) in Jackson County, AL, in 2015 (NWHC#44797-145). For comparison to European isolates of *P. destructans*, we used whole-genome data from isolates from the Czech Republic (isolates CCF3941, CCF3942, CCF4124, and CCF4125) available in the NCBI SRA Database (accession numbers SRR3411506, SRR3411507, SRR3411508, and SRR3411509), as well as the North American type isolate (NWHC#20631-21; NCBI SRA accession number SRR1952982).

Sequencing followed the manufacturer’s (Thermo Fisher Scientific) recommendations using the following kits: Ion plus fragment library kit (catalog number 4471252), Ion library TaqMan quantitation kit (catalog number 4468802), Ion PGM Hi-Q view OT2 (OneTouch 2) kit (catalog number A29811), Ion 318v2 (catalog number 4484354), and Ion PGM Hi-Q view sequencing kit (catalog number A30043). The raw sequencing data were processed using the default settings in the Torrent Suite software version 5.0.4. For each isolate, we obtained more than 12× average depth of sequencing coverage over the *P. destructans* genome reference. Single-nucleotide polymorphisms (SNPs) were identified from the next-generation sequencing reads using the Snippy pipeline version 3.0 (12). Briefly, reads were aligned to the *P. destructans* type isolate (NWHC#20631-021) reference genome (13) (GenBank accession number GCA_001641265.1) using Burrows-Wheeler Aligner (BWA) version 0.7.12-r1044 (14), and variants were called using FreeBayes version 0.9.21-7-g7dd41db (15) with a minimum read coverage of 4 (for each isolate, the genome coverage was at least 98.27% at a 4× threshold), minimum mapping quality set to 60, and minimum proportion for variant evidence set to 90%. A concatenated alignment of core SNPs (defined as an SNP that occurs at a genomic position present in all samples) was generated using Snippy, and an unrooted phylogenic tree was inferred from the resulting 13,379 characters using the neighbor-joining method and the Jukes-Cantor substitution model in CLC Genomics Workbench 9. As a secondary method, maximum-likelihood analysis was performed using the general time-reversible (GTR) substitution model in MEGA version 6.06.

**FIG 2:**
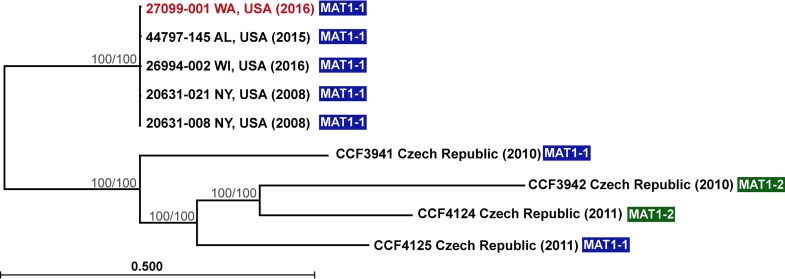
Phylogenetic relationships based on whole-genome sequence analysis of nine isolates of *Pseudogymnoascus destructans* from North America and Europe. A core alignment of 13,379 SNPs was generated for each isolate, using Snippy (11), and phylogeny was inferred by drawing an unrooted neighbor-joining tree supported with 1,000 bootstrap replicates (branch lengths represent the number of substitutions per site). A second analysis using maximum likelihood produced a tree with identical topology. Thus far, isolates of *P. destructans* from North America appear to be clonal, whereas there is considerable variation in populations of *P. destructans* in Europe (even in this small sample size of isolates from a limited geographical range). Isolates from both mating types (*MAT1-1* and *MAT1-2*) are found in Europe, suggesting that sexually recombining populations contribute to the increase in genomic variability. A single mating type (*MAT1-1*) is known from North America. Bootstrap values (neighbor-joining analysis/maximum-likelihood analysis) for well-supported nodes (>90) are presented.

The genome sequence of the isolate of *P. destructans* from Washington was statistically indistinguishable from the four North American isolates, forming a clade distinct from the four European isolates (Fig. 2). Although they were collected from a more confined geographic region (two hibernacula in the Czech Republic), the European isolates exhibited much higher genetic diversity than the North American isolates and included both known mating types; only the *MAT1-1* mating type has thus far been identified among isolates from North America (Fig. 2) (16). The minimal diversity and single mating type observed among isolates of *P. destructans* from the Nearctic is consistent with clonal spread of the pathogen in North America. These observations also suggest that the Washington isolate of *P. destructans* is likely of North American origin rather than representing an independent introduction event from Eurasia.

The timing and mechanism by which *P. destructans* may have reached the Pacific Northwest are unclear. The nearest detection of *P. destructans* is over 2,100 km away in eastern Nebraska. In the decade since *P. destructans* was first detected in North America, the fungus has spread approximately 1,900 km from the suspected introduction site. Thus, the recent detection in Washington appears inconsistent with the previously documented and predicted pattern of pathogen spread (17). Sequence analysis of the Washington bat’s mitochondrial NADH dehydrogenase subunit 1, cytochrome *b* (cyt*b*), and cytochrome *c* oxidase subunit I (COI) genes (18, 19) most closely matched GenBank sequences for little brown bats from western North America. Phylogenetic analyses performed on the cyt*b* and COI genes further demonstrated that the infected bat from Washington resided within a well-supported clade (bootstrap support values of 100 and 95 for the cyt*b* gene and the COI gene, respectively; see Fig. S1 in the supplemental material) that included members of *M. lucifugus* alascensis, a subspecies restricted in distribution to western North America. This finding suggests that the animal became infected with *P. destructans* in the Pacific Northwest and that the bat was not a vagrant from eastern North America, where WNS is now endemic. Sampling of bats and cave environments in the western United States since the winter of 2013-2014 without detection of the fungus suggests that the pathogen is not widespread or abundant in this environment in western states (i.e., over 2,000 samples tested from Arizona, California, Colorado, Idaho, Montana, Nebraska, New Mexico, Nevada, Oklahoma, Oregon, Texas, Utah, Washington, and Wyoming; A. Ballmann, unpublished data). More intensive surveillance efforts to define the range of *P. destructans* in western North America should improve our understanding of the presence, distribution, and origin of this pathogen in the Pacific Northwest.

10.1128/mSphere.00148-16.1 Figure S1  Phylogenetic trees from maximum-likelihood analyses of nucleotide sequences from bat mitochondrial genes. Sequences were aligned using MUSCLE in MEGA version 6.06, and phylogenetic analyses were conducted with RAxML-HPC2 version 8.2.4 (23) using the CIPRES Science Gateway (24). For both analyses, the general time-reversible model with gamma distribution was used with 1,000 bootstrap iterations. The analysis of the cytochrome *b* (cyt*b*) gene included a sampling of sequence data for various species of *Myotis* present in GenBank, while the analysis of the cytochrome *c* oxidase subunit I (COI) gene included sequence data generated from 148 *M. lucifugus* bats originating from across North America by Vonhof et al. (19). Both trees demonstrated strong support for the Washington bat with white-nose syndrome (denoted as NWHC 27099-1) residing within a clade representative of *M. lucifugus* alascensis. Data with support values of ≥85 are presented. Download Figure S1, PDF file, 0.3 MB. Copyright © 2016 Lorch et al. 2016 Lorch et al. This content is distributed under the terms of the Creative Commons Attribution 4.0 International license.

The presence of *P. destructans* in western North America has major implications for conservation of bat populations. The addition of a potential second disease epicenter could expose novel host species to the pathogen, accelerate the rate at which WNS spreads, and reduce the amount of time that wildlife management agencies have to develop mitigation strategies. Rapid responses to WNS in western North America may be difficult, because the locations of hibernation sites for many western bat species are unknown or inaccessible (20, 21). Furthermore, the wintering strategies of many species of bats in the Pacific Northwest are not well understood, with some species known to forage throughout the winter (22). Consequently, new approaches may be necessary to facilitate pathogen surveillance, monitor disease impacts, and conduct mitigation efforts for WNS in this region. The severity, magnitude, duration, and potential ecosystem-level effects of WNS in North America rank it among the most consequential wildlife disease events ever recorded. Although much progress has been made in understanding WNS and in monitoring its spread, more work is needed to determine how the disease dynamics and impacts vary among bat populations in eastern and western North America.

### Accession numbers.

Nucleic acid sequences for the NADH dehydrogenase subunit 1, cytochrome *b*, and cytochrome *c* oxidase subunit I genes of the little brown bat with WNS from Washington are available in GenBank (accession numbers KX290926, KX290927, and KX463942, respectively). Next-generation sequencing data are available through the NCBI SRA database (accession number SRP075419) and the BioProject database (accession number PRJNA322173).
