# Supplementary figures and images for: First Detection of Bat White-Nose Syndrome in Western North America
Source: mSphere. 2016 Aug 3;1(4):e00148-16. doi: 10.1128/mSphere.00148-16 (PMC4973635; doi:10.1128/mSphere.00148-16)

COI

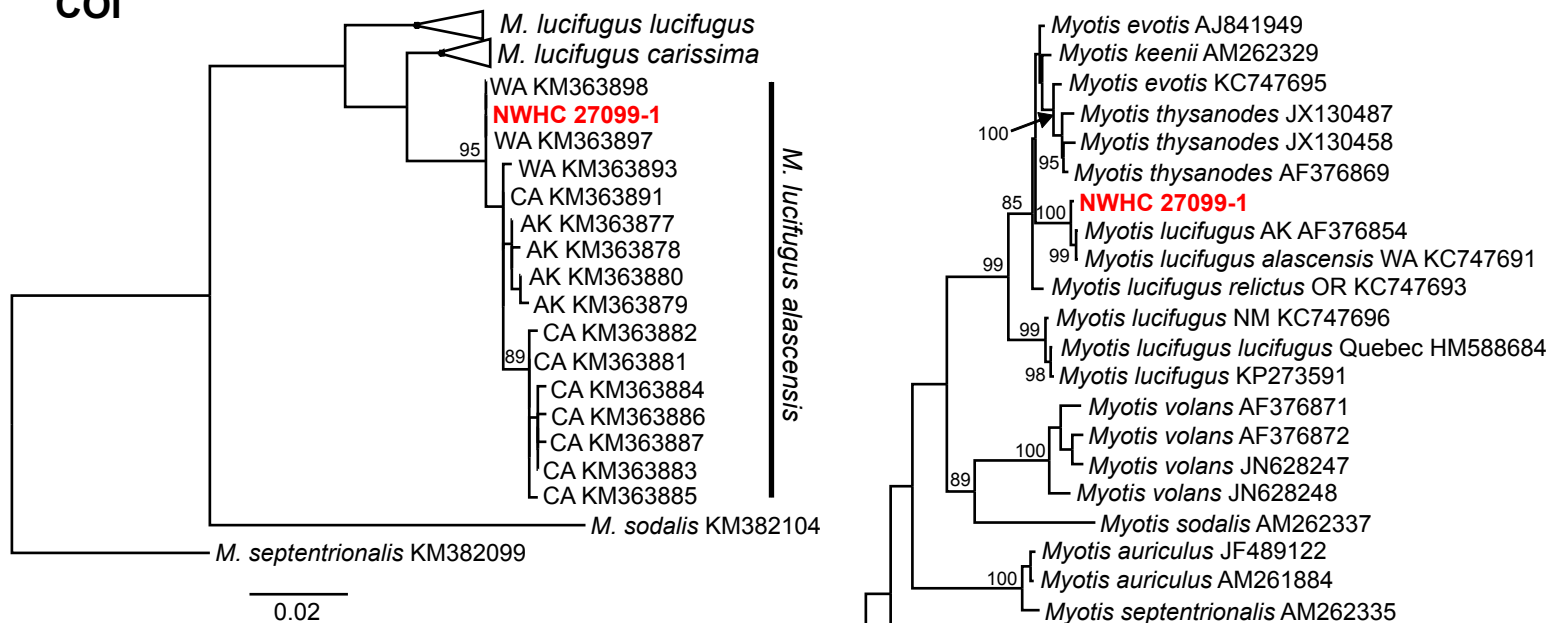

cytb

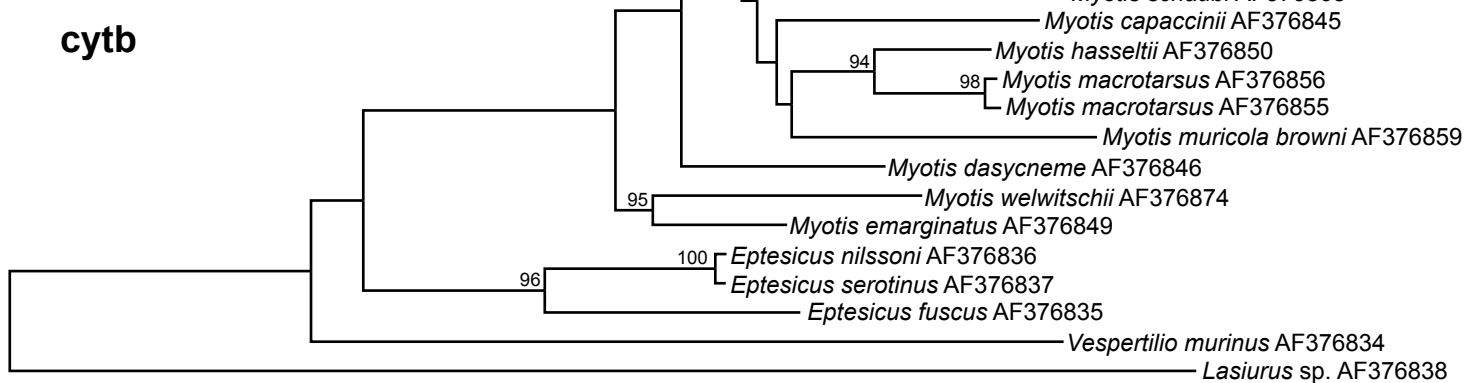

0.2

Supplement: Figure S1 [file sph004162125sf1.pdf]
